# Young Adults at the National Epicenter of the COVID-19 Outbreak: Understanding the Impact and Future Challenges of Social Distancing on Mental Health Outcomes

**DOI:** 10.3390/ijerph21010033

**Published:** 2023-12-26

**Authors:** Sana Malik, Ijeoma Opara, David T. Lardier, Jessica Younger, R. Neil Greene

**Affiliations:** 1 School of Social Welfare, Stony Brook University, Stony Brook, NY 11794, USA; jeyounger@institute.org; 2 Department of Social & Behavioral Sciences, Yale School of Public Health, Yale University, New Haven, CT 06520, USA; ijeoma.opara@yale.edu; 3 Department of Psychiatry and Behavioral Sciences, School of Medicine, The University of New Mexico, Albuquerque, NM 87131, USA; dalardier@salud.unm.edu; 4 Center on Alcohol, Substance Use And Addiction (CASAA), University of New Mexico, Albuquerque, NM 87131, USA; rngreene@unm.edu

**Keywords:** mental health, anxiety, depression, loneliness, young adults, COVID-19

## Abstract

Objectives. To understand the role and future implications of social distancing on mental health and substance use in young adults between the ages of 18 and 35 living in high-disease-prevalent areas of New York. Methods. Participants completed a self-administered online survey through Qualtrics. Results. 43.9% of the sample met criteria for moderate or severe anxiety, and 53.1% of the sample met criteria for moderate to severe depression. 76.1% of the sample experienced both anxiety and depressive symptoms. Despite this, the rates of lifetime mental health diagnoses, treatment, and access to mental health services were low. Rates of depression and anxiety differed across socio-demographic variables (gender, income, sexuality, education, and insurance status). Experiencing severe symptoms of the COVID-19 virus, poor coping skills, loneliness, increased alcohol use, and sleep disturbances were linked to higher rates of depression, anxiety, or both. Conclusion. As the first epicenter of COVID-19 in the United States, New York represents an important location for prevention researchers to learn about how COVID-19 affected a diverse population of young adults. It is essential that researchers and practitioners proactively develop early and appropriate interventions to address the ongoing mental health crisis and also plan for future crises.

## 1. Introduction

With the arrival of COVID-19 in the United States in March of 2020, New York became the epicenter of the virus due to high rates of transmission and deaths. As such, new regulations took place to contain the spread of the virus, including stay-at-home orders and social distancing. Common activities that support social connectedness became severely limited or banned in New York for three months, thus increasing risk factors for loneliness (defined as a discrepancy in desired and achieved social relationships). Additional behavioral changes and epidemic-related stressors were further predicted to impact well-being and mental health outcomes in all affected groups.

Disaster research models are being used to examine the health effects of COVID-19. Whether a pandemic fits into the structure of previous disaster research has yet to be determined, though some similarities have been reported, specific to impacts on individuals and communities [1,2]. The disaster literature points to determinants of vulnerability that include degree of exposure [1,3,4,5,6], destruction of property [7], bereavement, threat to life, physical injuries, and the individual’s behavior after the disaster [1,4]. The worst outcomes are found in those who have suffered greater personal loss and high levels of destruction [1]. In addition, vulnerable groups such as women [8], nonbinary folks [8], and people who are single [9] were found to have the highest levels of psychological distress.

Emerging or young adults (18–35), in particular, have been identified as the most likely to have severe impairment after a disaster [5,10] and are more likely to experience long-term health problems [1]. Such outcomes designate young adults as important to monitor, particularly single young adults [4] who represent 75% of all young adults aged 18 to 24 in the United States [11]. Recent investigations have indicated that being single was a risk factor for both negative postdisaster mental health [4] and COVID-19 mental health [9]. Inadequate psychosocial resources, including social support and healthy coping techniques, are additional risk factors [3,5] disproportionately impacting young adults who have the lowest medical insurance rate [11]. Adolescence and young adulthood are known as times of experimentation, especially with substances. This group demonstrates the highest rate of substance use of any other age group [11]. Predisaster substance use can worsen postdisaster outcomes [12] by unintentionally increasing use as a coping mechanism during a natural disaster or pandemic. Disasters alone can increase mental distress, which in turn can increase substance use [13] and influence long-term population health outcomes. Preliminary data from the COVID-19 pandemic demonstrate that young adults were vulnerable to negative mental health outcomes, including anxiety and depression [14]. Increases in social isolation led to negative impacts on work activities, nonwork activities, behavior, and overall wellbeing [14,15]. In addition, insomnia was found to mediate the relationships between social isolation and both anxiety and depression [16].

Previous disaster and pandemic research has found that the highest psychological distress is found in geographical areas most affected by the disaster or virus [17], and as such, young people in New York are at risk for negative mental health outcomes. Given that New York was the epicenter of the COVID-19 outbreak in the United States and provided the dearth of information available about the mental health implications of the novel coronavirus, this study aimed to fill the gap in knowledge about mental health impacts on young adult populations in New York. The researchers examined the potential consequences of stay-at-home orders, loneliness, negative coping, substance use, and sleep disturbances on mental health outcomes through an online, cross-sectional survey among young people, ages 18 to 35, living in New York State from July to September, 2020.

## 2. Methods

The study sample included (*N* = 684) participants who were surveyed using Qualtrics software. Eligibility criteria include the following: (1) being between the ages of 18 and 35; (2) residing in New York at the time stay-at-home orders were issued; (3) being able to read and understand English. The survey was disseminated from July to October of 2020, and recruitment occurred through social media channels, including email listservs, Facebook, and Twitter. Before proceeding to complete the survey, each participant consented to participate in this study by selecting a ‘yes’ response to the question, “Do you agree to participate in this study?” All (*N* = 684) participants received information about COVID-19 safety precautions and mental health resources at the end of the survey, and only the first (*N* = 270) received a USD 10 gift card due to funding restrictions. This study was approved by the Stony Brook University Institutional Review Board.

### 2.1. Measurement

#### Outcome Measures

Depression was measured using the Patient Health Questionnaire PHQ-9 [18,19]. The PHQ-9 is a nine-item self-report measure of depression in the previous two weeks before being surveyed and has been broadly validated among various demographic groups [15]. Sample items include the following: “Little interest or pleasure in doing things”. “Trouble falling or staying asleep, or sleeping too much”. The PHQ-9 was scored according to the following standard categorizations: mild (score of 5–9), moderate (score of 10–14), moderately severe (score of 15–19), or severe (>20) (M = 10.01, SD = 5.30, Cronbach’s Alpha = 0.84) [18].

Anxiety was measured using the Generalized Anxiety Disorder Scale GAD-7 [20]. The GAD-7 is a seven-item self-report measure of anxiety, with items focused on the previous two weeks prior to being surveyed [20]. Sample items include the following: “Feeling nervous, anxious, or on edge”. “Worrying too much about different things”. The GAD-7 was scored according to the following standard categorizations: mild (score of 5–9), moderate (score of 10–14), or severe (score > 15) (M = 8.56, SD = 4.44, Cronbach’s Alpha = 0.85).

### 2.2. Clinically and COVID-19 Relevant Indicators of Depression and Anxiety

COVID-19 related measures. Personal COVID-19 diagnosis was derived from a single item that asked participants to respond with no diagnosis (0), mild—symptoms effectively managed at home (1), moderate—symptoms severe and required brief hospitalization (2), and severe—symptoms severe and required ventilation. The majority of responding participants (73%) had no diagnosis of the COVID-19 virus, with 27% having mild or moderate symptoms. Family members diagnosed with the COVID-19 virus were assessed with a single open-ended question: “Number of extended family member(s) diagnosed with coronavirus”. Participant responses ranged from zero (0) to over 10 members of their family diagnosed with the COVID-19 virus (M = 2.51, SD = 2.33).

Coping during the COVID-19 pandemic was assessed using a single-item global coping question (question item: please rate how you feel you are coping with the COVID-19 pandemic on a scale ranging from 1 to 10). Responses were based on a 10-point Likert-type scale from not coping well at all (1) to coping extremely well (10). The mean response was 5.76 (SD = 1.81).

Loneliness was assessed using the three-item UCLA Loneliness Scale [21]. Sample items include the following: “How often do you feel that you lack companionship?” “How often do you feel left out?” Participant responses were collected using a three-item Likert-type scale from hardly ever (1) to often (3). Items were totaled to create a single loneliness variable (M = 5.77, SD = 1.44).

The increase in alcohol use during the COVID-19 pandemic was evaluated using a single item: “Has your drinking increased since New York issued stay-at-home orders?” Responses were collected dichotomously: yes (1) and no (0), with 54% of participants who drank indicating that their drinking has increased since the New York stay-at-home order.

Sleep severity was collected using the seven-item Insomnia Severity Scale [22]. This measurement tool examined participants’ perceptions of the current severity of insomnia/sleep-related problems using three items (e.g., difficulty falling asleep, difficulty staying asleep). Responses were collected using a five-point Likert-type scale from none (0) to very severe (4). A second set of four questions (e.g., How satisfied/dissatisfied are you with your current sleep pattern? How noticeable to others do you think your sleep problem is in terms of impairing the quality of your life?) examined if participants were satisfied/concerned/worried or distressed/felt sleep interfered with their daily functioning. Participant responses were also collected using a five-point Likert-type scale: (0) very dissatisfied/very worried/very noticeable/very much interfering (4). A total score for sleep severity was created by combining both of these subdimensions into one measurement variable (M = 18.69, SD = 5.43).

### 2.3. Covariates

Several sociodemographic covariates from the larger survey were tested as statistical controls. These covariates included age (in years), race–ethnicity, sexual orientation, years of education completed, employment status, and individual income. Participants were not obligated to respond to questions; therefore, missing information is present among sociodemographic variables.

### 2.4. Data Analysis

Data analyses were performed using STATA v. 15 [23] (StataCorp LLC., College Station, TX, USA). The Transparent Reporting of Evaluations with Nonrandomized Designs (TREND) statement was utilized as a guide for data analysis and reporting of results [24]. Baseline demographic data were assessed (see Table 1). The GAD-7 and PHQ-9 were scored according to standard guidelines [19,20]. 

The prevalence of anxiety and depression, by severity, was calculated and reported as percentages. Between-group difference tests (e.g., pairwise *t*-test with Bonferroni correction and analysis of variance (ANOVA)) were conducted using mean-level differences of depression, anxiety, and combined scores of depression–anxiety as outcome variables. Post hoc analyses using Tukey’s HSD test were calculated to identify the specific differences between groups. In Table 2 and Table 3, we report raw scores, mean differences, and statistical significance. Multivariate linear regression models were used to examine sociodemographic characteristics, clinically relevant indicators of depression and anxiety, and combined scores of depression–anxiety. Sociodemographic variables with a *p* ≤ 0.20 were chosen as covariates for linear regression analyses to determine which sociodemographic covariates were associated with depression and anxiety and the combined scores of depression–anxiety [25]. Traditional levels such as 0.05 can fail to identify variables known to be important [24]. Covariates were retained based on their meaningful contribution and statistical significance to the final analytical model [23]. We also examined all model variables to assure that all regression assumptions were met prior to the analyses [26]. Table 4 contains the six regression models, their beta (B) coefficients, adjusted beta coefficients, and 95% confidence intervals for beta coefficients.

## 3. Results

Table 1 presents the demographic characteristics of the study sample. The average age of participants was 27.95 ± 4.47 years. Most participants were 25 to 34 years of age (*n* = 494; 72.32%); female (*n* = 287; 42.02%); White (*n* = 305; 44.59%) or Hispanic, Latino, or Spanish (*n* = 138; 20.20%); and had some college (*n* = 142; 19.61%), a 2-year associate degree (*n* = 90; 13.15%), or a 4-year college or university degree (*n* = 183; 26.75%). The majority reported heterosexual sexual orientation (*n* = 454; 66.47%). Of those who responded, most received government or social assistance during COVID-19 (*n* = 165; 24.15%), were employed full-time at the time of being surveyed (*n* = 223; 32.65%) or part-time (*n* = 138; 20.20%), and had health insurance (*n* = 413; 60.46%).

### 3.1. Mental Health, Prior Diagnosis and Treatment, and Sleep Difficulties

Table 2 presents mental health scores, prior diagnoses, mental health treatment, and sleep difficulties. Among those who responded, 43.77% of the sample scored a GAD-7 of 10 or greater, an established indicator of moderate or severe anxiety symptoms in the last two weeks. On the PHQ-9, 53.10% of the sample scored a PHQ-9 score of 10 or greater, an established indicator of moderate or severe depressive symptoms in the last two weeks. Over three-fourths (76.1%) of participants experienced both anxiety and depression.

Despite the high prevalence of anxiety and depression symptoms, less than 10% of participants had a prior reported diagnosis of either condition. Only 57.54% of participants had received mental health treatment in their lifetime and 21.93% within the past 3 months prior to being surveyed. Participants reported high levels of sleep disturbances in the prior 2 weeks to being surveyed: 44.65% reported moderate to severe difficulties falling asleep, 40.84% had moderate to severe difficulty staying asleep, and 42.61% had moderate to severe problems with waking up too early.

### 3.2. Mental Health Scores by Socio-Demographic Characteristics

Depression and anxiety differed significantly across several socio-demographic variables (see Table 1). Depression, anxiety, and both depression–anxiety scores differed significantly by gender, income, sexuality, education, and insurance status. Depression and depression–anxiety scores differed by employment, with no difference noted solely for anxiety scores. Significant post hoc analysis differences were identified across income, sexuality, education, insurance status, and employment, with the exception of education for both depression and depression–anxiety scores.

### 3.3. Mental Health Scores by COVID-19 and Clinically Relevant Indicators

Depression, anxiety, and both depression–anxiety scores differed significantly on all COVID-19 and clinically relevant indicators (see Table 3). Significant pairwise *t*-tests were identified using Bonferroni correction. Significant post hoc analysis differences were identified across all ANOVA tests. Those with more severe symptoms of COVID-19 reported elevated scores of depression (16.25 ± 4.12), anxiety (12.45 ± 3.84), and both depression–anxiety (14.35 ± 3.82). Similarly, those reporting poorer coping skills during the COVID-19 lockdown in NYC reported elevated scores of depression (11.22 ± 4.92), anxiety (9.48 ± 4.47), and both depression–anxiety (10.36 ± 4.42). Reports of loneliness were related to higher mean scores of depression (14.51 ± 4.69), anxiety (12.01 ± 4.09), and both depression–anxiety (13.266 ± 3.92). Reports of increased alcohol use during the COVID-19 pandemic were indicative of elevated scores of depression (13.22 ± 5.06), anxiety (10.69 ± 4.50), and both depression–anxiety (12.07 ± 4.45). Last, severe sleep disturbances were attributable to higher mean scores of depression (18.00 ± 3.90), anxiety (15.07 ± 3.82), and both depression–anxiety (16.53 ± 3.67).

Table 4 reports multivariate linear regression analyses between sociodemographic covariates and COVID-19 and clinically relevant indicators on depression (PHQ-9), anxiety (GAD-7), and both depression–anxiety. COVID-19 diagnosis and clinically relevant indicators were positively associated with depression, anxiety, and both depression–anxiety. With depression, personal diagnosis of COVID-19 (β = 0.19, *p* < 0.01), loneliness (β = 0.25, *p* < 0.001), sleep disturbance severity (β = 0.40, *p* < 0.001), and increased drinking during COVID-19 had statistically significant associations (β = 0.10, *p* < 0.05). Positive coping during the COVID-19 lockdown in NYC was negatively associated with depression (β = −0.15, *p* < 0.01). Model 2 accounted for 34% of the variance in depression (R^2^ = 0.34, R^2^ Δ = 0.27, *p* < 0.001).

With anxiety, loneliness (β = 0.26, *p* < 0.001) and sleep disturbance severity (β = 0.32, *p* < 0.001) showed a statistically significant association. Similar to depression, positive coping during the COVID-19 lockdown in NYC was negatively associated with anxiety (β = −0.16, *p* < 0.01). Model 2 accounted for 36% of the variance in anxiety (R^2^ = 0.36, R^2^ Δ = 0.23, *p* < 0.001). Last, personal diagnosis of COVID-19 (β = 0.13, *p* < 0.05), loneliness (β = 0.27, *p* < 0.01), sleep disturbance severity (β = 0.38, *p* < 0.001), and increased drinking during the COVID-19 pandemic were statistically associated with depression–anxiety (β = 0.10, *p* < 0.01). Positive coping during the COVID-19 lockdown in NYC was negatively associated with depression–anxiety (β = −0.16, *p* = 0.001). Model 2 accounted for 40% of the variance in anxiety (R2 = 0.40, R2 Δ = 0.28, *p* < 0.001).

## 4. Discussion

The COVID-19 pandemic has had a significant effect on the physical and mental health of young adults. This research study sheds light on the impact of the COVID-19 pandemic on anxiety and depressive symptoms in young adults in New York. New York is a highly diverse state and international hub that has experienced a disaster within the past two decades that greatly impacted the physical and mental health of its residents. Disasters like the COVID-19 pandemic have been shown to directly and indirectly increase substance use, suicide, depression, and anxiety [1,3,4,5,6,8,13]. Often, there is a differential impact for people with pre-existing conditions (e.g., an increase in depressed mood for those with pre-existing alcohol problems) [12]. And unlike other natural disasters that tend to bring people together, COVID-19 precluded social interaction and support due to social distancing measures [24,27]. Social distancing measures due to the COVID-19 pandemic have been connected to social isolation, loneliness, and additional risks for negative mental health outcomes [25,28]. As the first epicenter of COVID-19 in the United States, New York represents an important location for prevention researchers to learn about how the COVID-19 pandemic affected young adults in order to develop sustainable prevention interventions for diverse populations.

This study demonstrated that COVID-19-related depression and anxiety symptoms were high for young adult populations across various demographics. Among all participants, 36.1% had mild anxiety, 33.8% had moderate anxiety, and 10.1% had severe anxiety. Furthermore, 29% of the sample had mild depression, 34.6% had moderate depression, 14.2% had moderate/severe depression, and 1.6% had severe depression. Those not employed and looking for work also had slightly higher scores of depression, anxiety, and both depression–anxiety when compared to others in the sample. Participants with no insurance reported slightly elevated scores of depression when compared to those with insurance.

Although studies point to women being at a greater risk for depression and anxiety [5,8], our study found that male participants reported slightly higher scores of depression (10.83 ± 4.80), anxiety (9.03 ± 2.01), and both depression–anxiety (10.04 ± 4.19). Norms related to masculinity and structural barriers to care may have contributed to challenges in accessing appropriate care for men during the pandemic. Greater COVID-19 impact, poorer coping skills, greater loneliness, higher alcohol use, and more severe sleep disturbances were also related to higher scores of depression, anxiety, and both depression–anxiety.

Consistent with trends in other COVID-19 studies, a majority of participants reported an increase in their use of alcohol after the stay-at-home orders were issued [29]. Findings from this study revealed that about 54% of participants reported an increase in alcohol use since the stay-at-home orders were issued; further analysis about substance use is presented in detail elsewhere [30]. This trend has been seen during other natural disasters and epidemics [31] as well. Chinese citizens who were exposed to the severe acute respiratory syndrome (SARS) pandemic in 2003 reported an increase in their drinking 1 year after the SARS pandemic was declared [31]. Natural and man-made disasters are also linked to long-term increases in drinking due to distress and exposure to traumatic events. Examples are the terrorist attacks that occurred in New York [32] and Hurricane Katrina on the Gulf Coast of the United States [33], which all reported an increase in drinking and other addiction-risk behaviors. This study found that increased alcohol use was associated with higher levels of depressive and anxiety symptoms. Similar trends were reported in previous studies, showing the association of anxiety symptoms following exposure to a traumatic event with alcohol use and misuse [34]. An explanation for this finding is the likelihood of young adults in the sample using alcohol as a coping mechanism to alleviate symptoms of anxiety and depression during the COVID-19 stay-at-home orders.

With the prevalence of negative mental health outcomes, the utilization of mental health services is crucial to the recovery of individuals and communities. Unfortunately, as was indicated in this study, there is discordance between the levels of need and treatment uptake, with only a small portion of the population receiving services after a disaster [35]. Factors that affect who receives services include predisposing characteristics, enabling resources, and perceived need [31]. A study conducted with the Chinese American population living in lower Manhattan after the World Trade Center attacks found that half the population had PTSD symptoms and only 4.4% saw a counselor [36]. More than half of those receiving treatment dropped out before the treatment was completed [35]. Specific to COVID-19, social distancing measures compromised the availability of mental health services and exacerbated pre-existing barriers in the US mental health system, including the limited accessibility of telehealth services, a lack of standardized insurance coverage for mental health services, poor population recognition of early signs of mental health distress, and mental health treatment stigma [37]. Given the extent of mental health concerns in the sample, it is evident that strengthening the nation’s mental health system is crucial to population health.

The mental health impacts of COVID-19 on young adults are likely to have far-reaching implications and present several challenges for the future, including long-term psychological effects that persist long after the pandemic, delayed developmental milestones due to restricted opportunities during the pandemic and beyond, increased demand for mental health services, long-term workplace challenges due to mental health challenges, and relationship challenges. Addressing these obstacles requires a multifaceted approach involving governments, educational institutions, healthcare systems, employers, and communities. It involves destigmatizing mental health issues, increasing access to mental health services, providing educational resources, and creating supportive environments for young adults to thrive despite the lingering impacts of the COVID-19 pandemic.

### Limitations

Strengths of this project include a diverse sample of New York residents, the use of previously validated scales, and further inclusion of other factors known to influence health behaviors and outcomes during disasters. In addition, these data provide early evidence of the mental health impacts of COVID-19 on young adult populations. However, several study limitations must be noted. First, given this was a cross-sectional sample, longitudinal and causal effects cannot be determined. Prospective longitudinal studies would offer several advantages in understanding the mental health impacts of COVID-19. Following individuals over time could provide researchers with crucial temporal perspectives for understanding how mental health is affected at different stages of the pandemic, from the initial outbreak to potential long-term recovery phases. Longitudinal studies could also minimize recall bias, as participants provide information in real-time rather than relying on retrospective recall, which can be subject to memory distortions. Finally, longitudinal studies can provide a clearer understanding of risk and protective factors, causal relationships, and individual and contextual factors that impact mental health in times of crisis and beyond and can be integrated into intervention design. Also, although online recruitment can cast a broader net than traditional recruitment and has been highly successful in recruiting hard-to-reach populations, such as young adults of racial/ethnic minorities and those with low education attainment, biases related to self-selection and sampling may be present [38]. As such, the sample may not be representative of and generalizable to the population of the state or the general population. This study provides important implications to consider for future mental health research, treatment, and prevention. Further longitudinal research is recommended to examine epidemiological conditions that reduce or aggravate mental health, including individual differences in responding to stressors and contextual factors such as changes in economic conditions, public health measures, and social support systems. Although this study focused on young adults, the health impacts of social distancing due to COVID-19 impacted all populations. As such, further research is recommended on other population groups, including children and the elderly, to determine if there are similarities and/or differences in the impacts of COVID-19 and the need for mental health interventions. In addition, further research is recommended to determine the format and content of interventions that are more effective for different population groups. Qualitative research may be helpful to elicit intervention design from target populations.

## 5. Conclusions

Particularly in light of chronic, high-stress situations such as the COVID-19 pandemic, it is essential that researchers and practitioners proactively develop early interventions in the likely inevitable event that another pandemic occurs and/or that social distancing measures are reinstated. This study has demonstrated that the effects of COVID-19 are not singular but rather interrelated and complex, as can be seen through the relationships between COVID-19 impact, sleep severity, and loneliness, among other factors, and mental health outcomes. As such, focus must be given to the provision of integrated mental health services. Findings from this study have identified initial risk and protective factors in mental health outcomes among young adults and can impact the long-term planning of policies for appropriate mental health services and effective public health strategies in emergency situations. Further research is recommended on other vulnerable groups affected by social distancing, including aging populations and children.

## Figures and Tables

**Table 1:** Mental health diagnosis during the COVID-19 pandemic by socio-demographics.

		Depression				Anxiety				Both Anxiety and Depression			
Demographics	*n*	Mean	SD	Difference Test (*t*-Test, *f*-Test)	*p*-Value	Mean	SD	Difference Test (*t*-Test, *f*-Test)	*p*-Value	Mean	SD	Difference Test (*t*-Test, *f*-Test)	*p*-Value
Full sample	684	10.00	5.30	---		8.55	4.44						
Age (years)				2.36	0.09 ^b^			0.29	0.75 ^b^			1.23	0.29 ^b^
18–24	157	9.27	5.79			8.45	4.79			8.84	5.03		
25–34	494	10.29	5.11			8.61	4.35			9.51	4.49		
35+	33	8.00	5.61			7.50	3.46			7.87	4.470		
Gender				2.92	0.004 ^a^			2.01	0.04 ^a^			2.63	0.009 ^a^
Male	211	10.83	4.80			219	9.02			10.00	4.19		
Female	287	9.43	5.58			288	8.22			8.89	4.86		
Race/Ethnicity				0.44	0.77 ^b^			0.46	0.76 ^b^			0.35	0.84 ^b^
American Indian/Alaskan Native	24	9.00	5.65			5.00	2.82			7.00	4.24		
Asian	4	10.31	6.38			9.02	4.84			9.67	5.41		
Black or African American	82	10.04	3.99			8.42	3.30			9.18	3.36		
Hispanic, Latino, or Spanish	138	10.44	5.12			8.59	4.38			9.59	4.48		
White	305	9.74	5.43			8.51	4.61			9.20	4.77		
Government/Social Service Financial Assistance				0.82	0.43 ^b^			0.68	0.51 ^b^			0.66	0.50 ^b^
No	26	11.19	3.89			9.46	3.12			10.42	3.32		
Yes	165	10.50	5.11			8.60	4.12			9.59	4.37		
In process	17	9.18	4.15			8.11	4.16			8.90	3.77		
Income				4.11	0.007 ^b^			7.71	<0.001 ^b^			5.455	0.001
Less than USD 20,000	76	8.53	5.72			7.86	4.91			8.20 *	4.90		
USD 20,000–USD 49,999	192	10.72 *	4.63			9.59 *	3.91			10.18 *	4.04		
USD 50,000–USD 99,999	196	9.62 *	5.56			7.61 *	4.58			8.71 *	4.89		
USD 100,000 +	31	11.18	4.86			9.35	3.62			10.33	3.91		
Sexuality				4.54	0.004 ^b^			5.95	0.001 ^b^			5.50	0.001 ^b^
Straight (heterosexual)	454	9.71 *	5.15			8.30 *	4.21			9.07 *	4.45		
Gay, lesbian, queer	16	13.12 *	4.88			11.12	6.04			12.12 *	4.81		
Bisexual	18	12.88	7.32			11.66 *	6.00			12.27 *	6.25		
Other/questioning	4	12.75	2.62			11.25	4.19			12.00	2.94		
Education				2.23	0.04 ^b^			2.35	0.03 ^b^			2.12	0.05 ^b^
High school graduate	28	8.32	4.96			8.21	5.18			8.26	4.77		
Some college	134	9.76	4.83			8.40	3.96			9.13	4.15		
2-year associate degree	90	11.02	4.94			8.58	3.58			9.77	4.19		
4-year college or university degree	183	9.79	5.58			8.20 *	4.73			9.11	4.89		
Some postgraduate or professional education	32	11.73	5.56			11.23	4.94			11.60	4.85		
Postgraduate or professional degree received	29	8.82	5.29			9.06 *	5.29			8.94	4.82		
Employment				2.62	0.02 ^b^			1.799	0.11 ^b^			2.39	0.04 ^b^
Employed, full-time	223	9.58 *	5.31			8.15	4.65			8.94 *	4.70		
Employed, part-time	138	10.14	5.37			8.73	4.44			9.52	4.67		
Not employed, looking for work	90	11.51 *	5.05			9.56	3.88			10.57 *	4.25		
Not employed, not looking for work	32	8.50	5.80			7.68	4.86			8.09	5.08		
Unable to work	21	8.52	3.29			8.00	2.70			8.26	2.80		
On unpaid leave	3	10.66	7.76			10.66	7.50			10.66	7.57		
Insurance				15.78	<0.001 ^b^			21.81	<0.001 ^b^			4.62	0.001
No	92	11.97 ^ᵻ^	4.80			10.47 ^ᵻ^	4.21			11.36 ^ᵻ^	4.17		
Yes	413	9.56	5.30			8.13	4.38			8.90	4.59		

^a^ Independent samples *t*-test. ^b^ ANOVA. * Significant post hoc analysis differences between groups are present in those cases in which superscript asterisks differ. ^ᵻ^ Significant pairwise *t*-tests were identified using Bonferroni correction.

**Table 2:** Mental health scores and responses (*N* = 683).

	*n*	%
Anxiety; GAD-7 Score; M = 8.56 (SD = 4.44)		
No (GAD-7 score < 10)	384	56.3
Yes (GAD-7 score of 10 or more)	299	43.7
Anxiety Severity		
No anxiety (<5)	137	20
Mild anxiety (5–9)	247	36.1
Moderate anxiety (10–14)	231	33.8
Severe anxiety (≥15)	68	10.1
Depression; PHQ-9 Score; M = 10.01 (SD = 5.30)		
No (PHQ-9 score < 10)	320	46.9
Yes (PHQ-9 score of 10 or more)	363	53.1
Depression Severity		
No depression (<5)	122	17.9
Mild depression (5–9)	198	29
Moderate depression (10–14)	236	34.6
Moderate/severe depression (15–19)	97	14.2
Severe depression (≥20)	30	1.6
Depression And Anxiety		
No symptoms (<5)	163	23.9
Both depression and anxiety symptoms (>5)	520	76.1
Mental Health Diagnoses (ever diagnosed)		
Depression	22	3.2
Anxiety	15	2.2
PTSD	1	0.1
Other mental health disorder	1	0.1
No diagnosis given	656	94.4
Mental Health Treatment (lifetime)		
Yes	393	32.8
No	290	67.2
Mental Health Treatment (Past 3 months)		
Yes	143	21.0
No	540	79.0
Sleep Patterns (last 2 weeks)		
Difficulty falling asleep (moderate to severe)	305	44.7
Difficulty staying asleep (moderate to severe)	279	40.9
Problems waking up too early (moderate to severe)	291	42.7

**Table 3:** Mental health diagnosis during the COVID-19 pandemic by coping, alcohol use, and sleep patterns.

		Depression				Anxiety				Both Anxiety and Depression			
	*n*	Mean	SD	Difference Test (*t*-Test, *f*-Test)	*p*-Value	Mean	SD	Difference Test (*t*-Test, *f*-Test)	*p*-Value	Mean	SD	Difference Test (*t*-Test, *f*-Test)	*p*-Value
All participants	608	15.18	40.15			23.35	43.74						
COVID-19 Diagnosis				37.44	<0.001 ^b^			19.28	<0.001 ^b^			3.83	<0.001
None (was not diagnosed)	376	8.86	4.93			7.82	4.32			8.41	4.35		
Mild (symptoms effectively managed at home)	85	11.44 *	4.60			9.56	4.00			10.54 *	4.05		
Moderate (symptoms severe and required brief hospitalization)	49	16.25 *	4.12			12.45 *	3.84			14.35 *	3.82		
Number of Family Members Diagnosed with COVID-19				8.80	0.002 ^b^			5.06	<0.001 ^b^			7.54	<0.001 ^b^
0	424	8.94	5.29			7.91	4.54			8.49	4.66		
1–5	244	11.25 *	5.05			9.35 *	4.23			10.36 *	4.38		
6–9	18	8.05	5.90			7.61	4.96			7.83	5.30		
10+	9	9.44	4.33			6.88	2.84			8.16	3.48		
Coping during COVID-19				8.44	<0.0011 ^b^			7.99	<0.001 ^b^			8.22	<0.001 ^b^
Coping poorly	143	11.22	4.92			9.48	4.47			10.36	4.42		
Coping moderately well	86	10.68	4.13			9.25	3.32			9.99	3.37		
Coping very well	280	9.11 *	5.67			7.83 *	4.62			8.56 *	4.95		
Loneliness				52.41	<0.001 ^b^			79.18	<0.001 ^b^			72.24	<0.001 ^b^
Hardly ever lonely	90	5.16	3.92			5.24	4.18			5.25	3.74		
Some of the time lonely	351	10.46 *	4.78			8.79 *	4.02			9.67 *	4.18		
Often lonely	60	14.51 *	4.69			12.01 *	4.09			13.26 *	3.92		
Alcohol Use Increase				−7.25	<0.001 ^a^			−5.34	<0.001 ^a^			−6.82	<0.001 ^a^
No	107	8.24	5.1			7.47	4.62			7.93	4.49		
Yes	123	13.22 ^ᵻ^	5.00			10.69 ^ᵻ^	4.50			12.07 ^ᵻ^	4.45		
Sleep Severity				39.25	<0.001 ^b^			66.34	<0.001 ^b^			60.85	<0.001
No issues	39	3.51	3.59			3.89 *	3.683			3.72	3.44		
Mild sleep disturbances	107	8.08 *	4.61			7.16 *	4.29			7.71 *	4.05		
Moderate sleep disturbances	154	10.94 *	3.81			9.35 *	3.27			10.18 *	3.28		
Severe sleep disturbances	43	15.55 *	3.95			11.53 *	3.34			13.54 *	3.14		
Very severe sleep disturbances	14	18.00*	3.90			15.07 *	3.83			16.53 *	3.67		

^a^ Independent samples *t*-test. ^b^ ANOVA. * Significant post hoc analysis differences between groups are present in those cases in which superscript asterisks differ. ^ᵻ^ Significant pairwise *t*-tests were identified using Bonferroni correction.

**Table 4:** Multivariate linear regression analyses between sociodemographic covariates and COVID-19 and clinically relevant indicators of depression, anxiety, and combined depression–anxiety.

	Depression						Anxiety						Depression and Anxiety					
Predictors	Model 1			Model 2			Model 1			Model 2			Model 1			Model 2		
	B (95% CI)	SE	β	B (95% CI)	SE	β	B (95% CI)	SE	β	B (95% CI)	SE	β	B (95% CI)	SE	β	B (95% CI)	SE	β
Gender (ref: female)	−1.51 (−3.06, −0.05) *	0.76	−0.13	−0.89 (−1.93, 0.13) *	0.52	−0.07	−1.23 (−2.43, −0.02) *	0.61	−0.12	−0.73 (−1.71, 0.258)	0.50	−0.07	−1.47 (−2.74, −0.20) *	0.64	−0.14	−0.85 (−1.76,0.04) *	0.45	−0.08
Sexual orientation Identification (ref: heterosexual)	1.43 * (0.01, 2.86)	0.72	0.13	0.84 (−0.19, 1.88)	0.52	0.07	1.80 (0.64, 2.97) **	0.59	0.19	1.10 (0.09, 2.11) *	0.51	0.11	1.57 (0.37, 2.77) **	0.60	0.16	0.93 (0.03, 1.84) *	0.45	0.10
Health insurance (ref: Yes)	−2.75 (−4.82, −0.67) *	1.05	−0.17	0.4 (−1.04, 1.93)	0.75	0.02	−3.56 (−5.24, −1.89)	0.84	−0.26	−1.45 (−2.98, −0.01) *	0.73	−0.11	−3.24 (−5.00, −1.49) ***	0.88	−0.24	−.63 (−1.95, 0.68)	0.66	−0.07
Personal diagnosis of COVID-19 (ref. No diagnosis)				1.50 (0.66, 2.34) **	0.42	0.19				0.52 (−0.30, 1.35)	0.41	0.07				0.91 (0.17, 1.65) *	0.37	0.13
Number of family members diagnosed with COVID-19				−0.43 (−1.30, 0.43)	0.44	−0.04				−0.57 (−1.41, 0.25)	0.42	−0.07				−0.58 (− 1.34, 0.16)	0.38	−0.03
Coping during COVID-19				−0.99 (−1.61, −0.36) **	0.31	−0.15				−0.92 (−1.51, −0.33) **	0.30	−0.16				−0.91 (−1.46, −0.37) **	0.27	−0.15
Loneliness				0.92 (0.52, 1.32) ***	0.20	0.25				0.82 (0.44, 1.20) ***	0.19	0.26				0.88 (0.53, 1.22) ***	0.17	0.27
Sleep severity				0.78 (0.55, 1.01) ***	0.116	0.40				0.53 (0.31, 0.75) ***	0.11	0.32				0.64 (0.53, 0.75) ***	0.10	0.38
Drinking increase during COVID-19				1.14 (0.88, 2.33) *	0.60	0.10				0.66 (−0.47, 1.80)	0.57	0.06				0.91 (0.59, 1.23) **	0.53	0.10
F value	5.55			42.23			7.70			20.16			8.13			37.11		
R^2^	0.07			0.34			0.13			0.36			0.12			0.40		
R^2^ Δ	0.07			0.27			0.13			0.23			0.12			0.28		
*p*-value	<0.001			<0.001			<0.001			<0.001			<0.001			<0.001		

* *p* < 0.05; ** *p* < 0.01 *** *p* < 0.001.

## Data Availability

Data is available upon request.
